# Biomineralization of Cu_2_S Nanoparticles by Geobacter sulfurreducens

**DOI:** 10.1128/AEM.00967-20

**Published:** 2020-09-01

**Authors:** Richard L. Kimber, Heath Bagshaw, Kurt Smith, Dawn M. Buchanan, Victoria S. Coker, Jennifer S. Cavet, Jonathan R. Lloyd

**Affiliations:** a Williamson Research Centre for Molecular Environmental Science, Department of Earth and Environmental Sciences, University of Manchester, Manchester, United Kingdom; b School of Biological Sciences, Faculty of Biology Medicine and Health, University of Manchester, Manchester, United Kingdom; University of Tokyo

**Keywords:** *Geobacter sulfurreducens*, copper, nanoparticles, Cu_2_S, bioreduction

## Abstract

Biomineralization of Cu has been shown to control contaminant dynamics and transport in soils. However, very little is known about the role that subsurface microorganisms may play in the biogeochemical cycling of Cu. In this study, we investigate the bioreduction of Cu(II) by the subsurface metal-reducing bacterium Geobacter sulfurreducens. Rapid removal of Cu from solution was observed in cell suspensions of G. sulfurreducens when Cu(II) was supplied, while transmission electron microscopy (TEM) analyses showed the formation of electron-dense nanoparticles associated with the cell surface. Energy-dispersive X-ray spectroscopy (EDX) point analysis and EDX spectrum image maps revealed that the nanoparticles are rich in both Cu and S. This finding was confirmed by X-ray absorption near-edge structure (XANES) and extended X-ray absorption fine structure (EXAFS) analyses, which identified the nanoparticles as Cu_2_S. Biomineralization of Cu_x_S nanoparticles in soils has been reported to enhance the colloidal transport of a number of contaminants, including Pb, Cd, and Hg. However, formation of these Cu_x_S nanoparticles has only been observed under sulfate-reducing conditions and could not be repeated using isolates of implicated organisms. As G. sulfurreducens is unable to respire sulfate, and no reducible sulfur was supplied to the cells, these data suggest a novel mechanism for the biomineralization of Cu_2_S under anoxic conditions. The implications of these findings for the biogeochemical cycling of Cu and other metals as well as the green production of Cu catalysts are discussed.

**IMPORTANCE** Dissimilatory metal-reducing bacteria are ubiquitous in soils and aquifers and are known to utilize a wide range of metals as terminal electron acceptors. These transformations play an important role in the biogeochemical cycling of metals in pristine and contaminated environments and can be harnessed for bioremediation and metal bioprocessing purposes. However, relatively little is known about their interactions with Cu. As a trace element that becomes toxic in excess, Cu can adversely affect soil biota and fertility. In addition, biomineralization of Cu nanoparticles has been reported to enhance the mobilization of other toxic metals. Here, we demonstrate that when supplied with acetate under anoxic conditions, the model metal-reducing bacterium Geobacter sulfurreducens can transform soluble Cu(II) to Cu_2_S nanoparticles. This study provides new insights into Cu biomineralization by microorganisms and suggests that contaminant mobilization enhanced by Cu biomineralization could be facilitated by *Geobacter* species and related organisms.

## Abstract (precis)

Dissimilatory metal-reducing bacteria are ubiquitous in soils and aquifers and are known to utilize a wide range of metals as terminal electron acceptors. These transformations play an important role in the biogeochemical cycling of metals in pristine and contaminated environments and can be harnessed for bioremediation and metal bioprocessing purposes. However, relatively little is known about their interactions with Cu. As a trace element that becomes toxic in excess, Cu can adversely affect soil biota and fertility. In addition, biomineralization of Cu nanoparticles has been reported to enhance the mobilization of other toxic metals. Here, we demonstrate that when supplied with acetate under anoxic conditions, the model metal-reducing bacterium Geobacter sulfurreducens can transform soluble Cu(II) to Cu_2_S nanoparticles. This study provides new insights into Cu biomineralization by microorganisms and suggests that contaminant mobilization enhanced by Cu biomineralization could be facilitated by *Geobacter* species and related organisms.

## INTRODUCTION

Dissimilatory metal-reducing bacteria are ubiquitous in soils and aquifers and are able to respire a wide range of metals coupled to the oxidation of organic or inorganic compounds (1, 2). These processes play an important role in the biogeochemical cycling of metals in soils and subsurface environments (3, 4). In addition, microbial metal reduction can be harnessed for catalytic applications (5, 6) and bioremediation purposes (7, 8). Species of *Geobacter* and *Shewanella* are among the most intensively studied metal reducers due to their presence in many soil and freshwater environments, their respiratory diversity, and the availability of genomic data (9–11). These organisms are able to utilize a range of metals as terminal electron acceptors, including Fe(III), Mn(IV), U(VI), Pu(VI), Cr(VI), Ag(I), Pd(II), and V(V) (3, 12–18). These processes typically involve *c*-type cytochromes, which facilitate electron transfer from the inner membrane to the periplasm and outer membrane, where many of these metals are reduced (19–21). Although the versatility of these metal reducers is well established, relatively little is known about their interactions with Cu.

Cu is a widely encountered trace element, with its distribution in soil affected by climate, geology, and soil properties (22). In addition, anthropogenic sources can lead to elevated Cu concentrations (23–26). Cu is an essential trace metal found in almost all forms of life; however, it can also be highly toxic by binding to proteins and inactivating enzyme function or by catalyzing Fenton chemistry to produce reactive oxygen species. Thus, at elevated concentrations, the inherent toxicity of Cu can limit plant growth, lowering crop yield and quality (27–29), and can decrease microbial community diversity and activity (30). Microbial processes play an important role in controlling the environmental fate of metals, potentially immobilizing them via redox changes, including bioreduction, or through sulfidation reactions (31). However, biomineralization has also been reported to enhance the mobilization of Cu and other cocontaminants through the formation of colloidal Cu nanoparticles (25, 32). For example, Hofacker et al. suggested *Clostridium* sp. was responsible for the Cu nanoparticle formation under soil reducing conditions; however, they were unable to directly observe Cu(II) reduction in cell suspensions of the *Clostridium* isolates (33). Therefore, further work is required to identify subsurface microorganisms which can potentially influence the biogeochemical behavior of this important micronutrient.

We demonstrated recently that Shewanella oneidensis is able to reductively precipitate Cu(0) nanoparticles from a Cu(II)-containing solution (6). Interestingly, the use of deletion mutants revealed that *c*-type cytochromes in the Mtr pathway, which is commonly used to reduce metals in *Shewanella* species, did not play a role in Cu(II) reduction. The as-prepared Cu nanoparticles were shown to be catalytically active toward a range of “click-chemistry” cycloaddition reactions, which have applications in the pharmaceutical industry (34).

Here, we investigate the fate of Cu(II) when supplied to cultures of another well-characterized model metal-reducing bacterium, the obligate anaerobe Geobacter sulfurreducens, in order to better understand its potential role in the biogeochemical cycling and the fate of Cu in contaminated soils and sediments, including the ability to produce Cu nanoparticles. Cu toxicity toward G. sulfurreducens was examined, and that bacterium's ability to bioreduce Cu(II) and remove the metal from solution was monitored using inductively coupled plasma atomic emission spectroscopy (ICP-AES). Any biomineralization products were analyzed using transmission electron microscopy (TEM), scanning transmission electron microscopy (STEM), energy-dispersive X-ray spectroscopy (EDX), X-ray absorption near-edge structure (XANES) analysis, and extended X-ray absorption fine structure (EXAFS) analysis.

## RESULTS

### Cu(II) toxicity toward G. sulfurreducens.

**FIG 1:**
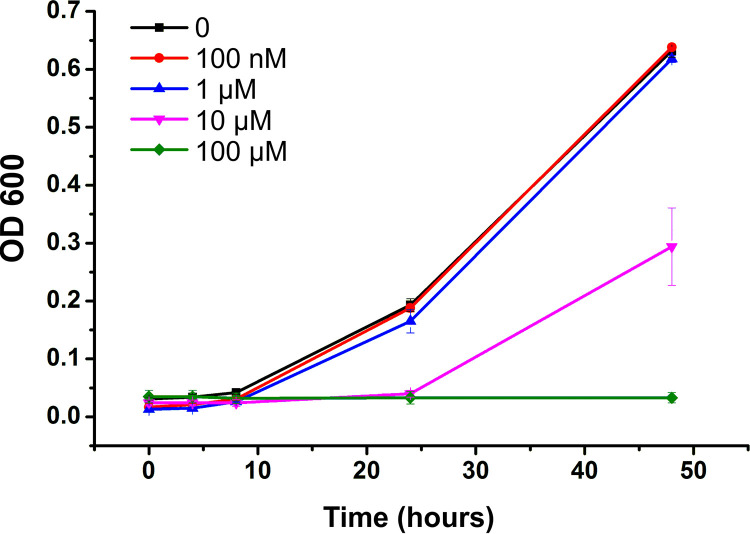
Anaerobic growth of G. sulfurreducens in minimal medium (NBAF) supplemented with different Cu(II) concentrations. Each concentration was performed in triplicate, with error bars representing the standard deviation of the replicates.

The effect of a range of concentrations of Cu on the anaerobic growth of G. sulfurreducens in minimal medium with lactate and fumarate supplied as the electron donor and acceptor, respectively, is shown in Fig. 1. Supplementation of the medium with 100 nM or 1 μM Cu(II) had little or no effect on the growth of G. sulfurreducens relative to a control with no added Cu(II). However, supplementation with 10 μM Cu(II) caused an extended lag phase with little to no growth seen over the initial 24 hours and only 45% growth seen after 48 hours relative to the no added Cu(II) control. With 100 μM Cu(II) supplementation, no growth was observed after 48 hours. Hence, under anoxic conditions, growth of G. sulfurreducens is strongly inhibited by Cu at concentrations of 10 μM and above.

### Removal of Cu(II) and formation of Cu nanoparticles.

**FIG 2:**
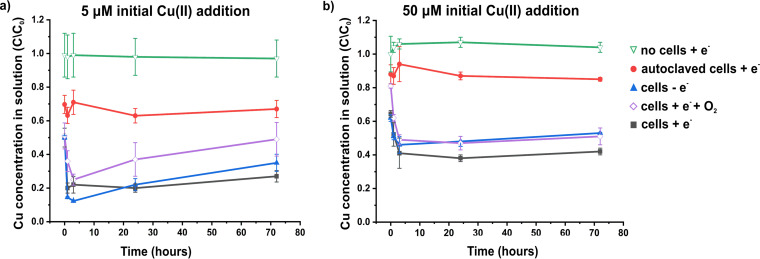
Concentration of Cu in solution in the presence of G. sulfurreducens when supplied with an initial Cu(II) concentration of 5 μM (a) or 50 μM (b). In both cases, Cu(II) was added to the medium prior to cell addition. The initial concentration of Cu was confirmed via ICP-AES. The first sampling time point (t1) was immediately after cell addition. Cu in solution was calculated as the concentration of Cu at a given time point (C) divided by the initial concentration prior to cell addition (C_0_), as determined by ICP-AES. Experiments were performed under anoxic conditions, except where indicated with the addition of O_2_ (purple diamonds). The addition or omission of acetate as an electron donor is indicated by +e^−^ or –e^−^, respectively. Each experiment was performed in triplicate, with error bars representing the standard deviation of these replicates.

Based on data from the toxicity assay and guided by Cu pore water concentrations in contaminated soils (25, 35), Cu(II) concentrations of 5 and 50 μM were chosen to be used in resting cell experiments to investigate the potential bioreduction of Cu(II) and the possible formation of lower oxidation state Cu nanoparticles, which was noted recently in cultures of other metal-reducing bacteria (6). Initial Cu(II) concentrations were confirmed via sampling of the solution prior to the addition of cells (t0). Decreases in the soluble Cu solution concentrations of 30.3% and 12.0% were observed in control experiments performed with dead (autoclaved) cells with initial concentrations of 5 μM and 50 μM Cu(II), respectively (Fig. 2). This decrease was observed at the first time point, taken immediately after inoculating the Cu(II)-containing medium with cells (t1), and then remained constant, consistent with initial Cu removal via rapid passive adsorption to the biomass. In the presence of live cells and an electron donor (acetate), a substantial further increase in Cu removal from solution compared with the dead (autoclaved) cell controls was observed, suggesting that most Cu removal in these treatments was facilitated by metabolic processes rather than by passive biosorption to the cell biomass (Fig. 2). When 5 μM Cu(II) was supplied, 50% Cu removal was observed immediately after addition of the cells (t1), with an increase to 80% removal at 1 hour. Cu removal remained stable at ∼80% at 24 hours, with a small decrease to 73% seen at the final 72-hour time point. When an initial concentration of 50 μM Cu(II) was supplied, only 36% of the Cu was removed by the live cells at the first time point (t1). Removal of Cu continued slowly for up to 24 hours, reaching a maximum of 63% removal at 24 hours before decreasing again slightly to 58% removal at 72 hours. Oxygenated cell controls (with acetate) showed decreased Cu removal at all time points compared with the anoxic cells when challenged with either 5 μM or 50 μM Cu(II). When no electron donor was supplied, initial Cu removal was slightly enhanced relative to the acetate-amended system when challenged with 5 μM Cu(II). However, after 3 h, Cu solution concentrations remained relatively stable in the acetate-amended experiment, but a significant increase in Cu solution concentrations was observed in both the electron donor-free and oxygenated cell controls. When the final sample was taken at 72 hours, Cu removal was greatest in the anoxic cells supplied with acetate for both the 5 μM and 50 μM Cu(II)-challenged systems.

**FIG 3:**
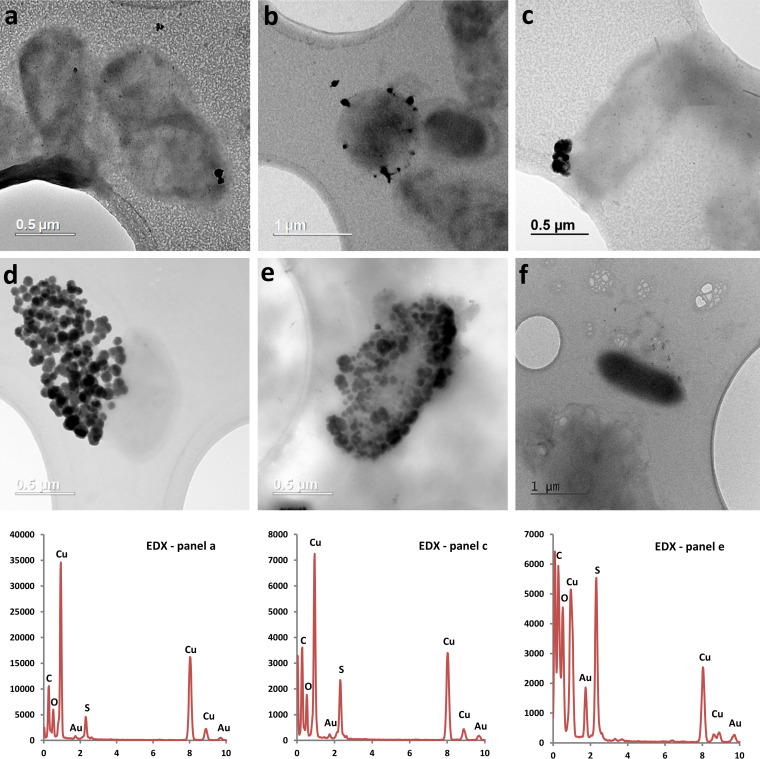
TEM images of G. sulfurreducens with associated Cu nanoparticles after being supplied with 5 μM Cu(II) (a to c) and 50 μM Cu(II) (d to f). The bottom row shows the corresponding EDX spectra of particles from panels a, c, and e (left to right). The *x* axis displays energy (keV), with the *y* axis displaying total counts. Samples for TEM imaging were taken at 24 h.

TEM images of samples taken at 24 hours demonstrated that when bacteria were supplied with acetate as an electron donor under anoxic conditions, removal of Cu by G. sulfurreducens resulted in the biomineralization of Cu nanoparticles (Fig. 3). These Cu nanoparticles were typically spherical and predominately associated with the cells, ranging in size from 10 to 90 nm.

**FIG 4:**
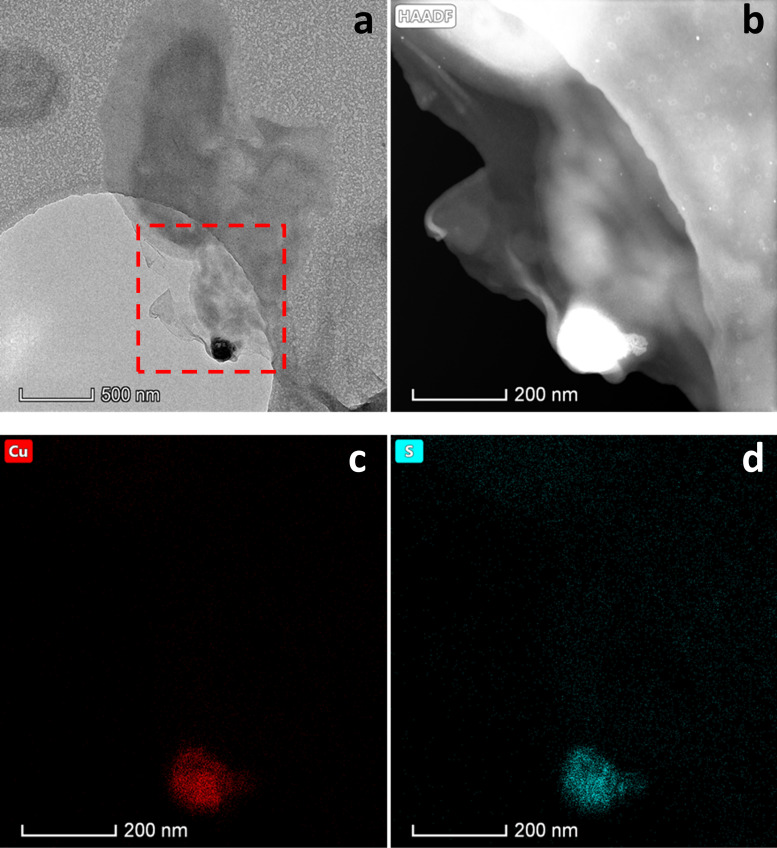
(a) TEM image of cells with Cu nanoparticles. (b) High-angle annular dark field (HAADF) image of the red dashed square from panel a. (c and d) EDX spectrum imaging of panel b, taken under STEM, showing Cu (c) and S (d).

EDX point analysis indicated that the particles were both Cu and S rich (Fig. 3). This finding was further supported by EDX spectrum imaging performed during STEM that revealed a close association between Cu and S in the nanoparticles (Fig. 4). A few particles were also seen in the dead (autoclaved) control; however, they were typically larger (100 to 200 nm) agglomerates and were far fewer than with live cells supplied with acetate. EDX analysis indicated these particles were also Cu and S rich (see Fig. S1 in the supplemental material). No nanoparticles were observed in the oxygenated cell control or no electron donor control (see Fig. S2 in the supplemental material).

### XAS characterization.

**FIG 5:**
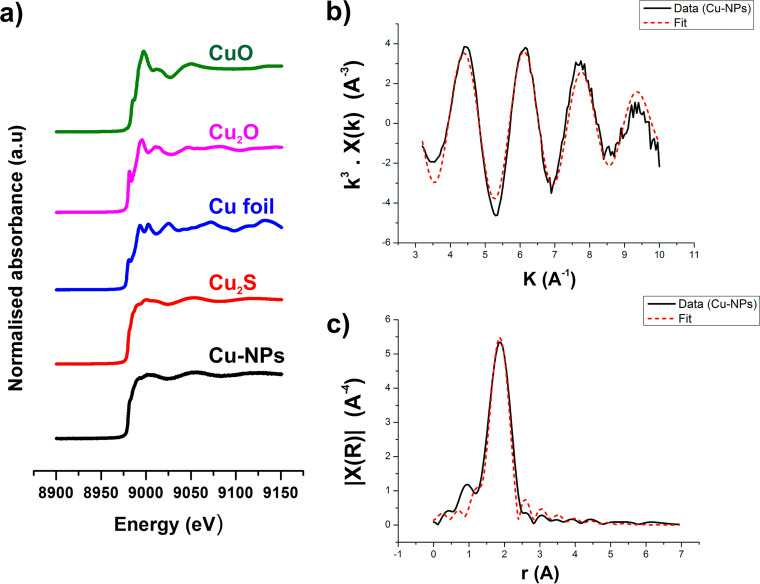
(a) XANES for the Cu K-edge of Cu nanoparticles produced by G. sulfurreducens (black line) and Cu standards. K^3^-weighted EXAFS data (b) and corresponding Fourier transform (c) for the Cu K-edge of the Cu nanoparticles (Cu-NPs). Data are shown by the black (solid) line, and the fit is shown by the red (dotted) line.

To identify the speciation of Cu and the local valence structure of the metallic nanoparticles, XAS characterization was performed on beamline B18 at the Diamond Light Source. The Cu K-edge X-ray absorption near-edge structure (XANES) spectrum collected on the Cu nanoparticles precipitated by G. sulfurreducens had an absorption edge energy (E_0_) of 8,980.4 eV. This aligned well with the E_0_ energy of a Cu_2_S (chalcocite) standard (8,980.1 eV). The excellent match between the XANES spectra for the Cu nanoparticles and the Cu_2_S standard clearly demonstrates that washed cell suspensions of the metal-reducing bacterium G. sulfurreducens catalyzed the formation of Cu_2_S particles (Fig. 5a). The oxidation state of Cu in Cu_2_S is thought to be dominated by Cu(I); however, the presence of significant Cu(II) and Cu(0) has also been suggested (36, 37). XANES data were identical whether the Cu nanoparticles were formed when the cells were supplied with Cu(II) as CuSO_4_ or CuCl_2_, ruling out any impact from the salt (data not shown).

Given the similarity between the Cu nanoparticles and Cu_2_S XANES spectra, the Cu nanoparticle EXAFS data were fitted assuming a coordination environment similar to that of the mineral chalcocite (Cu_2_S) (38). The best fit (Fig. 5b) was obtained with one shell of 3 S atoms at 2.30 ± 0.01 Å with a Debye-Waller factor of 0.009 ± 0.001. This is consistent with a Cu_2_S-like structure where Cu is coordinated by 3 S atoms at approximately 2.3 Å. Cu_2_S has 6 Cu atoms in the second shell; however, the Cu-S interatomic distance is poorly constrained (2.60 to 3.30 Å) and is likely to reduce the contribution from Cu scatterers to the EXAFS spectrum. Given this, the EXAFS data do not preclude the formation of a Cu_2_S phase. See Table S1 in the supplemental material for a complete description of the EXAFS fitting. Collectively, the XANES and EXAFS data support the biomineralization of the Cu(II) as poorly ordered Cu_2_S.

Cu L_2,3_-edge XAS was also performed on selected samples at the Advanced Light Source, Berkeley, CA. When live cells were supplied with an electron donor, peaks at 930.7 and 933.4 eV were observed (see Fig. S3 in the supplemental material). The peak at 930.7 eV is indicative of Cu(II), whereas the peak at 933.4 eV reflects the presence of Cu(I) (39, 40). Although the peak at 930.7 eV is qualitatively larger than the peak at 933.4 eV, the Cu(II) is known to be approximately 25 times more intense than the Cu(I) peak (39), suggesting a significant presence of Cu(I) in the sample and confirming that bioreduction took place. Cu L_2,3_-edge XAS data from the autoclaved and no electron donor controls also display peaks indicative of Cu(II) and Cu(I), although with a noticeable shift of +0.3 eV in peak positions. The intensities of the Cu(I) peaks are qualitatively smaller in both controls than in the live cells supplied with an electron donor, suggesting that less Cu bioreduction occurred in the controls.

## DISCUSSION

### Cu toxicity toward G. sulfurreducens.

Our results demonstrate that under anoxic conditions, concentrations as low as 10 μM Cu(II) strongly inhibit the growth of G. sulfurreducens. This value is over an order of magnitude lower than the Cu(II) concentrations (>100 μM) that are reported to be required to inhibit the growth of the enteric model organism Escherichia coli under similar conditions (anoxic chemically defined medium) (39). In a recent study, the model Fe(III)-reducing bacterium Shewanella oneidensis also appears to have greater resistance to Cu toxicity than G. sulfurreducens, with some growth of S. oneidensis still observed at 100 μM Cu in a similar chemically defined medium (6, 40), whereas growth of G. sulfurreducens is inhibited completely (Fig. 1). Hence, G. sulfurreducens appears to be particularly vulnerable to Cu toxicity.

### Biomineralization of Cu nanoparticles.

The relatively low tolerance of G. sulfurreducens toward Cu(II) compared with S. oneidensis is also reflected in their respective abilities to reduce Cu(II). In our previous study, a complete reduction of 50 μM and partial reduction of up to 200 μM Cu(II) to Cu(0) was observed for *S. oneidensis* (6). In the present study, G. sulfurreducens was capable of removing only up to 80% and 63% of 5 μM and 50 μM Cu(II) from solution, respectively. No removal at 200 μM Cu(II) was observed (data not shown). Our data suggest that Cu removal from solution is greatest in the presence of live cells, suggesting that microbial metabolism increases the removal of Cu from solution compared with biosorption by dead biomass. The highest removal was seen when live cells were supplied with an electron donor under anoxic conditions, with TEM and XAS data indicating that formation of Cu nanoparticles was significant only under these conditions. This result suggests that the presence of an electron donor and anoxic conditions are required for significant biosynthesis of Cu nanoparticles. Our characterization of these nanoparticles as Cu_2_S is supported by EDX point analysis, EDX mapping, XANES, and EXAFS data. As no nanoparticles were seen in the oxygenated or electron donor-free controls and XAS data support limited reduction to Cu(I) species, we attribute the observed removal of Cu from solution under these conditions to biosorption of Cu(II) to the cell surface and/or intracellular accumulation of the metal. Interestingly, the Cu solution concentrations in the oxygenated and electron donor-free controls were found to increase after 3 hours, but they remained relatively stable under anoxic conditions with an electron donor (acetate). We suggest that the rerelease of Cu into solution observed in the oxygenated and electron donor-free controls may be due to the export of intracellular Cu and/or desorption of cell-bound Cu, which was limited in the anoxic cells supplied with acetate due to immobilization of Cu via bioreduction and Cu_2_S precipitation.

The formation of Cu_2_S nanoparticles here is surprising, as previous work on Cu biomineralization by anaerobic bacteria found that, in the absence of sulfate-reducing conditions, nanoparticles formed were metallic Cu or Cu oxides (6, 25, 41, 42). Several mechanisms have been proposed for the formation of Cu(0) nanoparticles among different bacteria. Ramanathan et al. suggested that intracellular reduction and cellular efflux systems may play a role in the synthesis of extracellular Cu(0) nanoparticles by Morganella morganii (42). *Clostridium* species were implicated in the formation of Cu(0) nanoparticles in a flooded Cu-contaminated soil via cellular efflux of Cu(I), followed by disproportionation to Cu(II) and Cu(0) (25, 33). Conversion of the Cu(0) nanoparticles to Cu_x_S in the flooded soil was observed following the onset of sulfate reduction. As G. sulfurreducens is unable to carry out dissimilatory sulfate reduction, this mechanism cannot account for the formation of Cu_2_S nanoparticles seen here. Identical XANES spectra from Cu nanoparticles formed when G. sulfurreducens was supplied with Cu(II) as CuSO_4_ or CuCl_2_, further supporting a mechanism which does not directly involve sulfate reduction. Clearly, a different mechanism is responsible for the biomineralization of Cu_2_S nanoparticles in G. sulfurreducens than that for the Cu(0) nanoparticles typically produced by other bacteria studied to date.

Cell-bound thiol sites have been shown to dominate metal(oid) complexation in a range of microorganisms, including G. sulfurreducens (43, 44). In addition, intracellular Cu(I) is known to target Fe-S clusters in Escherichia coli under anoxic conditions, with Cu(I) displacing the Fe (45–47). Ligation of Cu with these sulfur groups could potentially play a role in the formation of Cu_x_S nanoparticles in organisms which are unable to reduce Cu(II) to Cu(0), such as G. sulfurreducens. However, elucidating the mechanisms of Cu nanoparticle formation in metal-reducing bacteria requires further work and will be the target of future studies.

This study demonstrates that G. sulfurreducens, a metal-reducing bacterium, is able to produce Cu_2_S nanoparticles from aqueous Cu(II). These results provide direct evidence of Cu biomineralization by a ubiquitous subsurface microorganism, which may suggest a role for this organism in the biogeochemical cycling of Cu and potential mobilization of cocontaminants in soil systems (25). Cu_x_S nanoparticles have previously been reported to form only following the onset of sulfate reduction. G. sulfurreducens is unable to respire sulfate, suggesting that biomineralization of Cu_x_S nanoparticles could also occur in the absence of sulfate-reducing conditions. In addition to the biogeochemical implications discussed above, biomineralization of Cu nanoparticles offers a promising green method for the production of Cu catalysts. We have previously demonstrated that Cu(0) nanoparticles produced by S. oneidensis are active click-chemistry catalysts (6). Cu_2_S nanoparticles also have many catalytic applications, including as electrocatalysts for oxygen evolution and CO_2_ reduction (48, 49). Therefore, tailored Cu nanoparticle catalysts could potentially be produced using specific microorganisms.

## MATERIALS AND METHODS

### Geobacter sulfurreducens.

All cultures of G. sulfurreducens (ATCC 51573) were grown anaerobically in a fully defined, presterilized, liquid minimal medium (NBAF) (50) at pH 7.1. Serum bottles (100 ml) containing NBAF were inoculated with a late-log/early-stationary-phase culture to give an optical density at 600 nm (OD_600_) of 0.02. The cultures were grown for 24 h at 30°C. Late-log cultures were transferred under anoxic conditions to centrifuge tubes, and the cells were pelleted by centrifugation at 4,960 rpm for 20 min at 4°C. The cells were washed two times with fresh anoxic, sterilized morpholinepropanesulfonic acid (MOPS) buffer (50 mM) and then were resuspended in the same buffer at pH 7.1.

### Toxicity assay.

Cells were grown in minimal medium as described above. Late-log-phase aliquots were used to inoculate 50 ml of anoxic minimal medium to give a starting OD_600_ of 0.015. Cu (CuSO_4_) was added from a stock solution to give a final concentration of 1 nM or 0.1, 1, 10, or 100 μM Cu(II). A Cu-free control was also used. Incubation was carried out at 30°C. Samples were taken under anoxic, aseptic conditions at 0, 4, 8, 24, and 48 h, and the optical density was measured to determine growth. All assays were performed in triplicate with a standard deviation of less than 0.012 between OD_600_ readings.

### Cu removal experiments.

The removal of Cu(II) by G. sulfurreducens was determined using pregrown and washed “resting cell” cultures supplied with excess electron donor. The cultures contained either 5 μM or 50 μM Cu(II) as CuSO_4_ (unless stated otherwise) and 30 mM sodium acetate as the electron donor in 50 mM MOPS adjusted to pH 7.1. The medium was purged with a gas mixture of N_2_:CO_2_ (80:20) for 20 minutes to remove O_2_, sealed in containers with thick butyl rubber stoppers, and autoclaved. Washed late-log/early-stationary-phase cells were then added aseptically to achieve a final OD_600_ of 0.2, and incubation was carried out at 30°C. Soluble Cu was determined by taking aliquots and centrifuging them at 14,900 × *g* for 10 minutes to pellet the cells and insoluble Cu. Samples were taken from the supernatant and Cu in solution measured using ICP-AES. All experiments were performed in triplicate.

### TEM imaging.

All sample preparations were performed under anoxic conditions in an anaerobic cabinet. A total of 1 ml of cell suspension from the Cu reduction assay (5 and 50 μM) was taken after 24 hours and centrifuged at 14,900 × *g* for 10 minutes; afterward, the supernatant was discarded and the pellet resuspended in 1 ml deionized water. A total of 1.5 μl of the cell suspension was pipetted onto a gold TEM grid with a carbon-coated Formvar or holey-carbon support film and air dried in an anaerobic chamber. Samples were kept anoxic until they were transferred into the TEM chamber. TEM imaging and EDX analysis were performed in an FEI TF 30 field emission gun (FEG) analytical TEM operated at 300 kV and equipped with an Oxford X-Max 80 windowless silicon drift detector (SSD) EDS system. EDX analysis was performed with the sample tilted at the optimum angle toward the detector to increase collection efficiency.

### STEM imaging.

STEM imaging and EDX analysis were performed in an FEI Talos F200A analytical transmission electron microscope (AEM) with an X-FEG electron source operated at 200 kV. High-angle annular dark field (HAADF) STEM imaging was performed using a probe current of approximately 250 pA. EDX analysis was performed using a Super-X four silicon drift detector EDX system with a total collection solid angle of 0.7 srad; all four detectors were turned on, and the sample was not tilted.

### XAS characterization.

For XAS characterization at the Cu K-edge, 1-ml aliquots of the G. sulfurreducens Cu reduction (5 and 50 μM CuSO_4_ and CuCl_2_) assays were taken and centrifuged at 14,900 × *g* for 10 minutes. The supernatant was discarded and the pellet resuspended in 1 ml anoxic deionized water. The sample was centrifuged again and resuspended in 1 ml anoxic deionized water before 200 μl of the suspension was pipetted onto a plastic weighing boat and air dried. Samples were mounted onto a layer of Kapton tape which in turn was mounted onto an aluminum sample holder. A further layer of Kapton tape was applied over the samples to maintain anaerobicity. X-ray absorption fine structure (XAFS) spectra were collected at the Cu K-edge (∼8,980 eV) at room temperature on beamline B18 at the Diamond Light Source. A 36-element solid-state Ge detector with digital signal processing for fluorescence XAFS, high-energy resolution, and high count rate was used to measure with the beam at 45° incidence with respect to the sample holder plane. All spectra were acquired in quick-EXAFS mode, using the Pt-coated branch of collimating and focusing mirrors, an Si(111) double-crystal monochromator, and a pair of harmonic rejection mirrors.

XAS processing and background subtraction were carried out using Athena, whereas EXAFS data were modeled using Artemis (Demeter 0.9.24 [51]). Fitting was calculated using multiple k-weights (k, k^2^, and k^3^) and the best fit was calculated in R space by minimization of the reduced χ^2^. At no point did the parameterization utilize more than two-thirds of the independent points available.

Cu L_2_,_3_-edge spectroscopy was performed at the Advanced Light Source, Berkeley, CA, on beamline 6.3.1.1. For sample preparation, 1-ml aliquots were taken and centrifuged at 14,900 × *g* for 10 min. The supernatant was discarded and the pellet resuspended in 1 ml anoxic deionized water. The sample was centrifuged again before final resuspension in 1 ml anoxic deionized water. A total of 200 μl of the suspension was dried as a powder onto a carbon sticky pad before being placed on an aluminum sample holder and stored under anoxic conditions prior to analysis. All manipulations were performed in an anaerobic chamber. X-ray absorption spectra were collected in total-electron yield (TEY) mode and normalized to the incident beam intensity.

## Supplementary Material

Supplemental file 1
