# Supplementary material for: Biomineralization of Cu2S Nanoparticles by Geobacter sulfurreducens
Source: Appl Environ Microbiol. 2020 Sep 1;86(18):e00967-20. doi: 10.1128/AEM.00967-20 (PMC7480366; doi:10.1128/AEM.00967-20)
Supplement: Supplemental file 1 [file AEM.00967-20-s0001.pdf]

Supporting Information

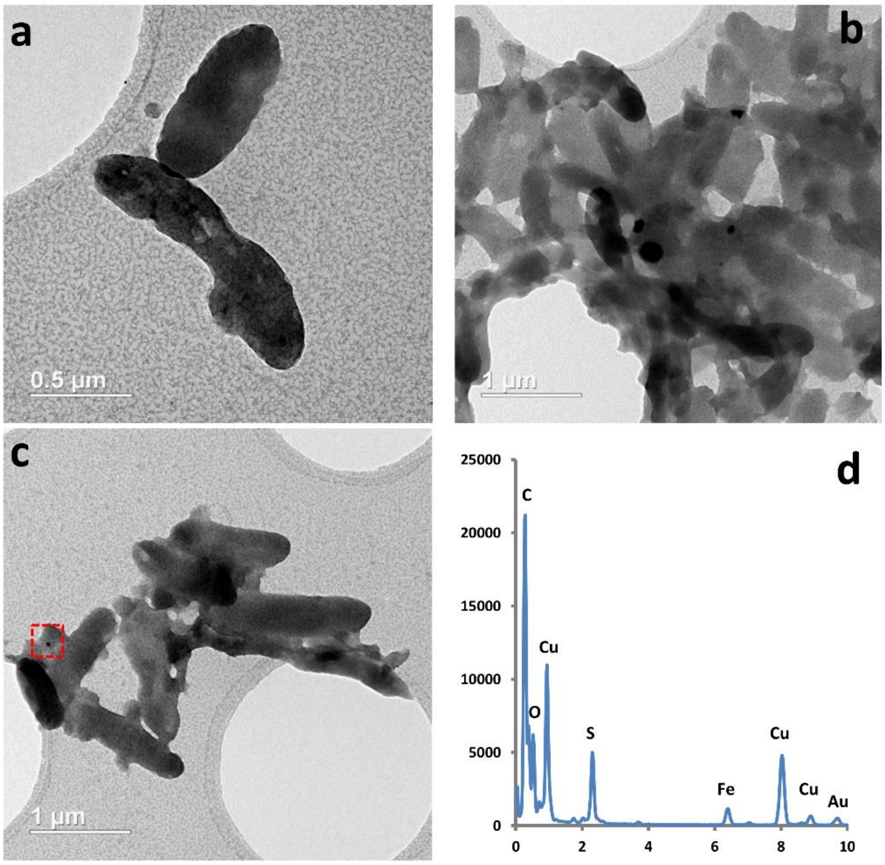

Figure S1 TEM images of autoclaved cells of *G. sulfurreducens* (a-c). Most cells showed no Cu nanoparticles present, however a number of larger agglomerates were seen. (d) EDX point analysis of large electron dense particle highlighted by red dashed square in (c).

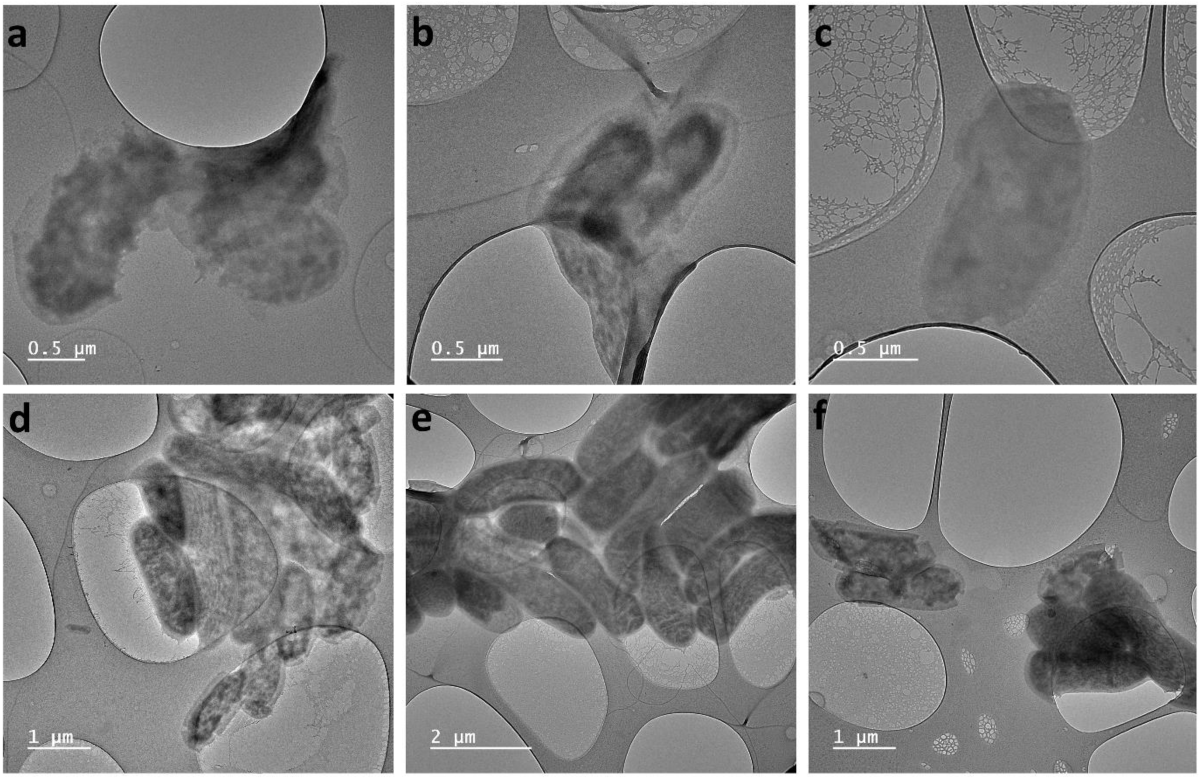

Figure S2 TEM images of no electron donor controls (a-c) and oxygenated cells with electron donor (d-f). No electron dense areas corresponding to Cu nanoparticles were observed.

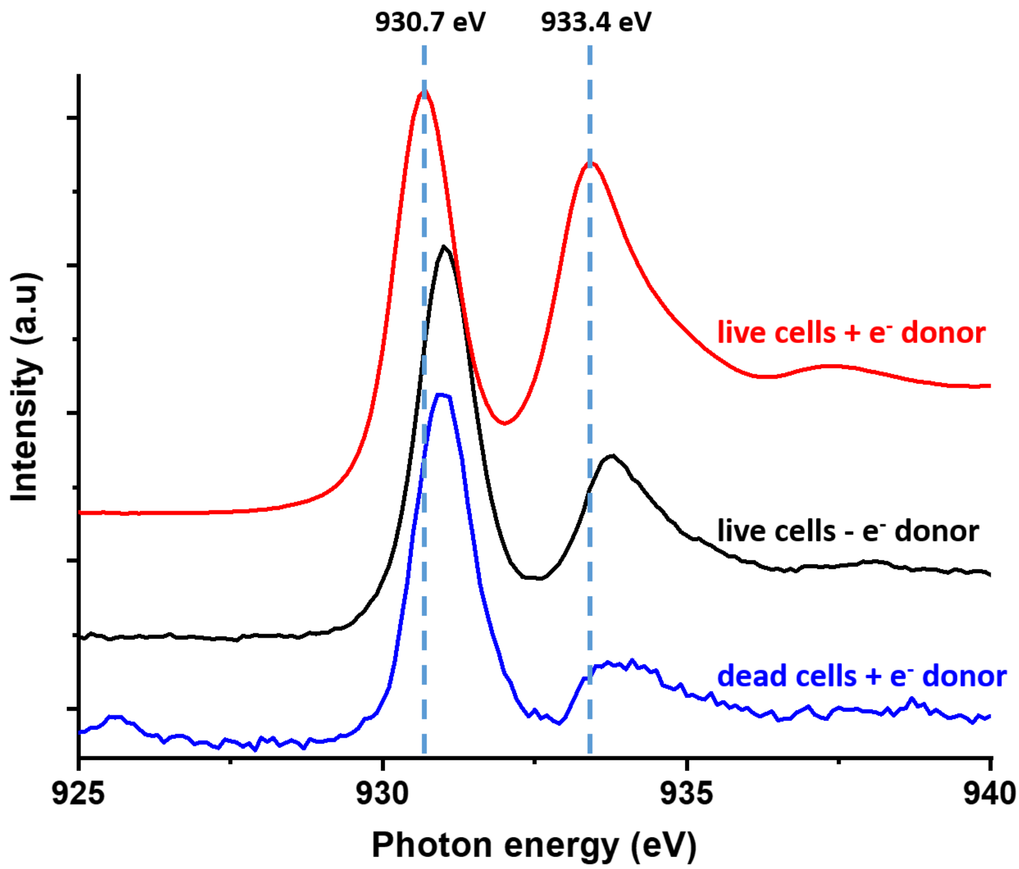

Figure S3 $L_{2,3}$-edge data for cells plus electron donor (top); cells with no electron donor control (middle); and heat killed (autoclaved) cells plus electron donor control (bottom).

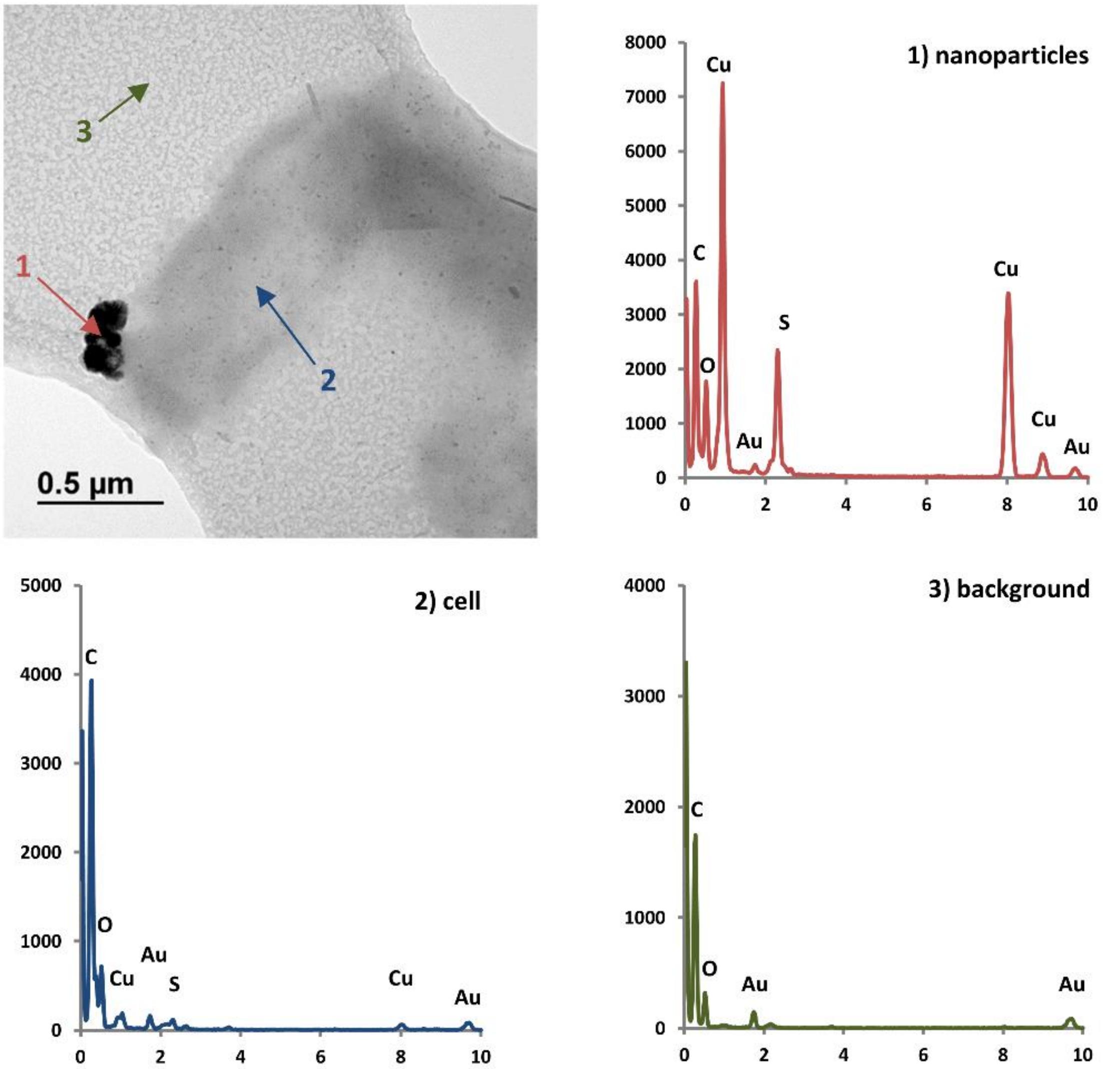

Figure S4 TEM image from Figure 3 (panel a) with additional EDX point analysis to compare EDX spectrum from electron dense area (1) to the bulk cell (2) and TEM grid background (3).

**Shell**	***N***	***R* [Å]**	**σ^2 [Å^2]**
Cu-S	3	2.301 ± 0.003	0.0097 ± 0.0013

S_0^2	0.90
ΔE_0	7.02
Reduced χ^2	808.0
R	0.03

Table S1. EXAFS best fit parameters for Cu nanoparticles produced by *G. sulfurreducens*
